# Blackbox: Psychosoziale Fälle in der Notaufnahme

**DOI:** 10.1007/s00063-022-00981-x

**Published:** 2023-01-12

**Authors:** Alina Ruegenberg, Martina Schmiedhofer, Anika Kreutzberg, Cornelia Henschke, Martin Möckel, Anna Slagman

**Affiliations:** 1 Notfallmedizinische Versorgungsforschung, Notfall- und Akutmedizin, Campus Virchow Klinikum (CVK), und Charité Campus Mitte (CCM), Charité – Universitätsmedizin Berlin, Charitéplatz 1, 10117 Berlin, Deutschland; 2 Fachgebiet für Management im Gesundheitswesen, Gesundheitsökonomisches Zentrum Berlin, Technische Universität Berlin, Berlin, Deutschland

**Keywords:** Psychische Erkrankungen, Drogenmissbrauch, Soziale Belastungen, Notfallversorgung, Soziale Notfallmedizin, Mental disorders, Substance abuse, Social problems, Emergency care services, Social emergency medicine

## Abstract

**Ziel der Studie:**

Ziel der Studie ist a) die Prävalenzerfassung psychosozialer Notfälle in der Notaufnahme, b) die Ermittlung des Anteils der nicht als (Neben‑)Diagnose kodierten psychosozialen Fälle (Dunkelziffer) und c) die Charakterisierung identifizierter Patient*innen.

**Methodik:**

In einer retrospektiven Studie wurden psychosoziale Notfälle einer Kalenderwoche aus der Routinedokumentation der zentralen Notaufnahme (ZNA) der Charité – Universitätsmedizin Berlin, Charité Campus Mitte (CCM) identifiziert. Nach Ausschluss von geplant aufgenommenen Fällen wurden 862 Patient*innen in die Studie eingeschlossen. Die identifizierten psychosozialen Notfälle wurden hinsichtlich ihrer soziodemografischen und klinischen Merkmale deskriptiv analysiert und mit anderen Notfällen verglichen.

**Ergebnisse:**

Die Prävalenz psychosozialer Notfälle unter Notaufnahmepatient*innen im angegebenen Zeitraum betrug 11,9 % (*n* = 103). Ein Großteil der psychosozialen Notfälle war in den Diagnosen nicht (35,9 %) oder nicht vollständig (20,4 %) kodiert. Es gab einen statistisch relevanten Unterschied in der Geschlechterverteilung mit einem signifikant höheren Männeranteil unter den psychosozialen Notfällen (70,9 %) im Vergleich zu anderen Notfällen (50,7 %; *p* < 0,0001). Die 2 häufigsten Behandlungsanlässe unter den psychosozialen Notfällen waren Substanzmissbrauch (66,0 %) und Obdachlosigkeit (20,4 %).

**Schlussfolgerungen:**

Diese Studie zeigt einen relevanten Anteil an in den Routinedaten dokumentierten psychosozialen Behandlungsanlässen an allen Behandlungsanlässen in der Notaufnahme und einen hohen Anteil von in den kodierten Diagnosen nicht erfassten Fällen (Dunkelziffer) auf. Notaufnahmen stellen somit eine wichtige Anlaufstelle für vulnerable Patient*innengruppen dar, die dort bisher nicht ausreichend identifiziert werden.

## Abstract (en)

**Objectives:**

The aims are (a) assessment of the prevalence of psychosocial emergencies in the emergency department (ED), (b) determination of the proportion of cases not coded as diagnosis (unreported cases), and (c) characterization of identified patients.

**Methods:**

In a retrospective study, psychosocial emergencies in one week were identified from routine documentation of the central ED of the Charité – Universitätsmedizin Berlin, Charité Campus Mitte (CCM). After exclusion of planned admitted cases, 862 patients were included in the study. The identified psychosocial emergencies were descriptively analyzed with regard to their sociodemographic and clinical characteristics and compared with other emergencies.

**Results:**

The prevalence of psychosocial emergencies in the reported period was 11.9% (*n* = 103). A large proportion of psychosocial emergencies were not coded (35.9%) or not fully coded (20.4%) as an ICD diagnosis (unreported cases). There was a statistically relevant difference in gender distribution with a significantly higher proportion of males among psychosocial emergencies (70.9%) compared to other emergencies (50.7%; *p* < 0.0001). The two most common treatment causes among psychosocial emergencies were substance abuse (66.0%) and homelessness (20.4%).

**Conclusions:**

This study shows a relevant proportion of psychosocial emergencies among all treatments in ED routine data and a high proportion of cases not captured in the coded diagnoses. EDs thus represent an important point of contact for vulnerable patient groups but standardized screening and identification are still lacking.

## Hintergrund und Fragestellung

Rettungsdienst und Notaufnahmen fungieren, auch wegen ihrer permanenten, barrierefreien Verfügbarkeit, als soziale „Sicherheitsnetze“ der Gesellschaft [1, 11]. Dies wird sichtbar in der Assoziation zwischen einem niedrigen sozioökonomischen Status und psychiatrischen Erkrankungen [27] sowie einer erhöhten Inanspruchnahme der Notaufnahmen bzw. des Rettungsdiensts dieses Personenkreises [7, 18, 27].

Weiterhin zeigt sich eine steigende Tendenz des Auftretens sog. psychosozialer Notfälle. Damit werden Behandlungsanlässe in der Notaufnahme beschrieben, die einerseits eine „durch eine soziale Mangelsituation getriggerte Exazerbationen einer psychischen Erkrankung“ [17] darstellen oder ausschließlich durch soziale Umstände wie beispielsweise Obdachlosigkeit bedingt sind [11, 14, 17, 22]. Als Ursache für eine steigende Inzidenz psychosozialer Notfälle wird ein Zusammenhang zu sich ändernden sozioökonomischen Ressourcen und Lebensumständen der Patient*innen genannt [17, 24]. Für die prähospitale Versorgung zeigen Auswertungen, dass psychosoziale und psychiatrische Notfälle den zweithäufigsten Einsatzgrund unter den Notarzteinsätzen darstellen [18]. Ambulant behandelte Fälle in Notaufnahmen aufgrund von ‚Faktoren, die den Gesundheitszustand beeinflussen und zur Inanspruchnahme des Gesundheitswesens führen‘ (sog. Z‑Diagnosen gemäß ICD10), wiesen zwischen 2009 und 2015 mit 214,7 % die vierthöchste Steigerungsrate unter allen Diagnosekapiteln auf [28]. Dies schließt Personen mit potenziellen Gesundheitsrisiken aufgrund sozioökonomischer oder psychosozialer Umstände ein.

Flächendeckende detaillierte Untersuchungen zur Prävalenz von psychosozialen Notfällen in Notaufnahmen in Deutschland sowie international sind bisher nicht vorhanden. Konkrete Daten zu einzelnen Standorten in Deutschland lassen auf einen Anteil zwischen 11,5 % [24] und 14–20 % [11] schließen. Personen, die unter der Armutsgrenze leben oder die Notaufnahmen aufgrund sozialer Notlagen häufig nutzen, zeigen zudem eine deutlich höhere Mortalitätsrate [10, 15, 16]. Für Patient*innen bedeutet dies eine inadäquate Versorgung, für überfüllte Notfallaufnahmen eine zusätzliche Belastung und für soziale Sicherungssysteme eine ineffiziente Versorgung, die langfristig mit erhöhten Kosten einhergeht. Als zentraler Auflaufpunkt dieser vulnerablen Personengruppen könnten Notaufnahmen einen wesentlichen Beitrag zur Identifikation dieser Patient*innen leisten. Durch konsequente Weiterleitung in geeignetere Versorgungsstrukturen wäre eine nachhaltige Verbesserung ihres sozialen und gesundheitlichen Zustands erreichbar.

Internationale Studien zeigten bereits, dass eine Intervention durch Sozialarbeiter*innen oder ein gezieltes Fallmanagement die Inanspruchnahme der Notaufnahmen durch diese Patient*innen reduzieren kann [2, 9, 23].

In der folgenden Arbeit wird primär die Prävalenz psychosozialer Notfälle unter den Notaufnahmefällen der zentralen Notaufnahme des Campus Charité Mitte durch ein retrospektives Screening der gesamten Notaufnahmedokumentation erfasst. Sekundär wird die erfasste Prävalenz mit der anhand von kodierten Diagnosen ermittelten Prävalenz verglichen, um daraus die Dunkelziffer psychosozialer Notfälle in der Routinedokumentation von Notaufnahmen abzuschätzen. Um mögliche Unterschiede von Patient*innen mit psychosozialen Behandlungsanlässen hinsichtlich ihrer soziodemografischen und klinischen Merkmale zu identifizieren, werden diese Fälle zudem mit anderen Notfällen verglichen.

## Studiendesign und Untersuchungsmethoden

### Studienpopulation

Alle Erste-Hilfe-Scheine (EH-Scheine) von Patient*innen, die in der Woche vom 06.05.2019 bis zum 12.05.2019 in der zentralen Notaufnahme am Charité Campus Mitte der Charité – Universitätsmedizin Berlin vorstellig wurden, wurden retrospektiv ausgewertet. Insgesamt wurden 867 Fälle aus dieser Woche bewertet. Ausgeschlossen wurden lediglich geplant in die Klinik aufgenommene Fälle (*n* = 5). Für die verbleibenden 862 Fälle wurde die im Folgenden beschriebene Datenerhebung durchgeführt.

### Variablen/Zielgrößen

Zur Definition der „psychosozialen Notfälle“ wurden die in Tab. 1 aufgeführten Kriterien, die aus der deutschsprachigen Literatur zu diesem Thema entnommen und um weitere Indikationen ergänzt wurden, verwendet [11, 17]. Das Vorliegen der genannten Kriterien wurde anhand der Dokumentation in der Notaufnahme überprüft. Ein Notfall wurde als psychosozial klassifiziert, sobald eines der Kriterien für die Definition eines psychosozialen Notfalls in der Notaufnahme erfüllt war. Die Bewertung erfolgte anhand der in der klinischen Routine dokumentierten Daten durch medizinisches Personal. Eine Goldstandarddiagnostik und ein prospektives Screening wurden nicht durchgeführt.

**Table 1:** 

Folgen von Substanzmissbrauch
Angst- und Panikstörungen
Belastung: akute Belastungsreaktion und Überforderung aufgrund psychischer oder sozialer Umstände
Suizidgedanken bei fehlender psychiatrischer Grunderkrankung
Familiäre Konfliktsituationen und Gewalt
Antisoziales Verhalten und Delinquenz
Pathologische Trauerreaktion
Folgen von Trennung
Verelendung, Armut
Pflegenotstand
Vereinsamung
Verwahrlosung
Obdachlosigkeit
Konfliktsituationen und Gewalt in der Partnerschaft
Arbeitslosigkeit^a^
Kontaktanlässe mit Bezug auf das Wohnumfeld/die wirtschaftliche Lage [13]
Stuprum bzw. Vergewaltigung
Opfer körperlicher Gewalt/Verbrechen

^a^ Nur als psychosozialer Notfall gewertet, sofern es einen weiteren psychosozialen Begleit- oder Hauptanlass gab

Zusätzlich zu den psychosozialen Notfällen wurden psychiatrische Notfälle gesondert erfasst. Als psychiatrischer Notfall wurde der Behandlungsanlass gewertet, wenn das behandelnde Notaufnahmepersonal eine entsprechende psychiatrische Diagnose gestellt hatte.

Die identifizierten psychosozialen Notfälle wurden in Begleit- oder Hauptanlass eingeteilt. Wenn ein psychosozialer Notfall als Begleitanlass gewertet wurde, wurde zusätzlich erfasst, welchen psychiatrischen oder somatischen Hauptanlass es gab.

Soziodemografische und klinische Charakteristika wurden für alle psychosozialen Notfälle aus der klinischen Routinedokumentation in der Notaufnahme extrahiert.

Die deskriptive statistische Auswertung erfolgte mit IBM SPSS® Statistics 24 (IBM, Armonk, NY, USA). Es wurden relative und absolute Häufigkeiten für kategoriale Variablen sowie Median und Interquartilsabstände (IQR) für metrisch skalierte Merkmale berechnet. Für den Vergleich der Gruppen „psychosozialer Notfall“ und „kein psychosozialer Notfall“ wurde für das Alter der Mann-Whitney-U-Test und für das Geschlecht der χ^2^-Test verwendet. Dabei wurde ein *p*-Wert mit *p* < 0,05 als statistisch signifikant gewertet.

Die retrospektive Datenverwertung für dieses Projekt wurde durch die Ethikkommission der Charité – Universitätsmedizin geprüft und positiv beschieden (EA1/082/18). Die Studiendurchführung erfolgte in Übereinstimmung mit der Deklaration von Helsinki.

## Ergebnisse

### Prävalenz psychosozialer und psychiatrischer Behandlungsanlässe in der Notaufnahme

**Figure 1:**
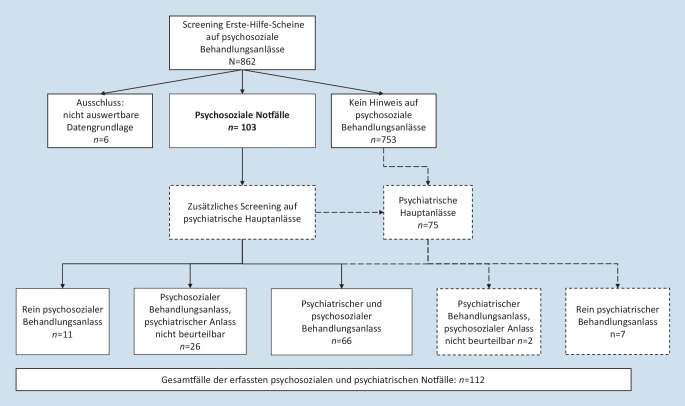

Von den 862 gescreenten Notaufnahmefällen wurden 103 (11,9 %) als psychosoziale Notfälle identifiziert, bei 753 (87,4 %) gab es keinen Hinweis auf psychosoziale Behandlungsanlässe (Abb. 1). Damit konnten 856 abschließend bewertet werden, bei den 6 nichtbewertbaren Fällen (0,7 %) waren die Angaben im Klinikinformationssystem unzureichend. Dies war beispielsweise der Fall, wenn Patient*innen die Notaufnahme bereits vor dem Arztkontakt wieder verlassen hatten. Neben den psychosozialen Notfällen wurden psychiatrische Hauptanlässe (*n* = 75 ≙ 8,7 %) aus allen Behandlungsanlässen gesondert erfasst. In 7,6 % (*n* = 66) der Fälle lag sowohl ein psychiatrischer als auch ein psychosozialer Behandlungsanlass vor. Es gab 7 rein psychiatrische Notfälle und 11 rein psychosoziale Notfälle. In den restlichen 2 psychiatrischen Notfällen und den restlichen 26 psychosozialen Notfällen war unbekannt, ob sie gleichzeitig auch einen psychosozialen bzw. einen psychiatrischen Notfall darstellten. Der Gesamtanteil der erfassten psychosozialen und psychiatrischen Notfälle entsprach damit 12,9 % (*n* = 112). Darunter waren am häufigsten die Diagnosen F10 bis F19 „Psychische und Verhaltensstörungen durch psychotrope Substanzen“ [13] vertreten.

### Vergleich demografischer Daten zwischen psychosozialen und anderen Notfällen

Die Geschlechterverteilung zwischen den psychosozialen Notfällen und den restlichen Fällen ergab einen statistisch signifikanten Unterschied (*p* < 0,0001): Bei den psychosozialen Notfällen überwog der Männeranteil mit 70,9 % (*n* = 73), bei den nichtpsychosozialen Notfällen waren mit einem Männeranteil von 50,7 % (*n* = 382) beide Geschlechter ungefähr gleich verteilt (Abb. 1).

Für das Alter ergab sich eine ähnliche Verteilung für psychosoziale Notfälle (medianes Alter: 42; IQR: 32–55; *n* = 103) und nichtpsychosoziale Notfälle (medianes Alter 43; IQR: 31–63; *n* = 753). Damit gab es keinen signifikanten Unterschied zwischen diesen beiden Gruppen (*p* = 0,319).

### Dunkelziffer psychosozialer Notfälle

In 35,9 % (*n* = 37) der Fälle wurden die psychosozialen Notfälle zwar in der Anamnese oder der körperlichen Untersuchung erwähnt, jedoch nicht mittels ICD-10 kodiert. Von den psychosozialen Notfällen waren zusätzlich 20,4 % (*n* = 21) unvollständig kodiert. Psychische Vorstellungsgründe wie Substanzmissbrauch oder Panikstörungen wurden mit je 51,5 % und 70,0 % häufiger vollständig kodiert als soziale Umstände wie beispielsweise Obdachlosigkeit (23,8 %). Bei psychiatrischen und gleichzeitig psychosozialen Notfällen (*n* = 66) waren im Vergleich zu rein psychosozialen Notfällen etwas mehr Fälle, insgesamt die Hälfte (*n* = 33 ≙ 50,0 %), vollständig kodiert.

### Charakterisierung psychosozialer Notfälle

**Figure 2:**
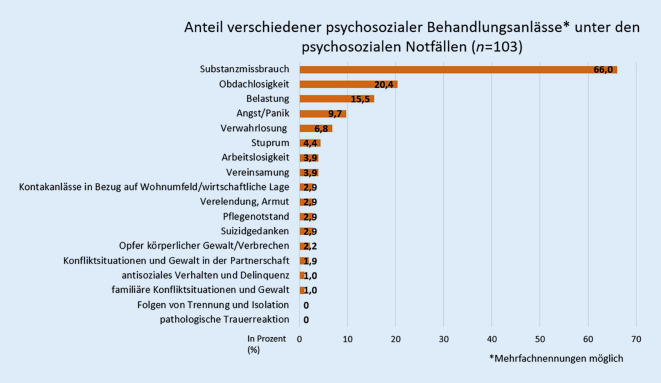

Der häufigste Behandlungsanlass unter den psychosozialen Notfällen war Substanzmissbrauch (66,0 %), gefolgt von Obdachlosigkeit (20,4 %) und akuten Belastungsreaktionen (15,5 %) bzw. Überforderung, Angst- und Panikstörungen (9,7 %) und Verwahrlosung (6,8 %; Abb. 2), dabei war die Vergabe mehrerer Behandlungsanlässe pro Fall möglich (Mehrfachnennung). Die weiteren erfassten Behandlungsanlässe waren nur vereinzelt vertreten. Die Behandlungsanlässe „pathologische Trauerreaktionen“ und „Folgen von Trennung und Isolation“ waren in den untersuchten Fällen nicht vertreten. Bei Frauen waren die Behandlungsanlässe Substanzmissbrauch (*n* = 17 ≙ 56,7 %) und Obdachlosigkeit (*n* = 4 ≙ 13,3 %) im Vergleich zu Männern (Substanzmissbrauch: *n* = 52 ≙ 71,2 %; Obdachlosigkeit: *n* = 17 ≙ 23,3 %) etwas seltener, Stuprum (w: *n* = 3 ≙ 10 %; m: *n* = 1 ≙ 1,4 %), Pflegenotstände (w: *n* = 3 ≙ 10 %; m: *n* = 0) und akute Belastungsreaktionen bzw. Überforderung (w: *n* = 7 ≙ 23,3 %; m: *n* = 9 ≙ 12,3 %) waren bei Frauen hingegen etwas häufiger.

Die Tab. 2 fasst weitere Charakteristika psychosozialer Notfälle zusammen. In nahezu der Hälfte aller Fälle (*n* = 103) stellte der psychosoziale Notfall den Hauptanlass (48,5 %) des Notaufnahmebesuchs dar, in der anderen Hälfte war er lediglich Begleitumstand (51,5 %). Beim größten Anteil (34,0 %) dieser Begleitanlässe war eine psychiatrische Erkrankung als Hauptbehandlungsanlass angegeben, gefolgt von traumatischen (22,8 %) und internistischen Hauptanlässen (20,9 %).

**Table 2:** 

	Ausprägungen	Absolute Werte (relative Werte in %)
Geschlecht	Männlich	73 (70,9)
	Weiblich	30 (29,1)
Behandlungsanlässe (häufigste)	Substanzmissbrauch	68 (66,0)
	Obdachlosigkeit	21 (20,4)
	Belastung	16 (15,5)
Psychosozialer Notfall als Vorstellungsgrund	Hauptanlass	50 (48,5)
	Begleitanlass	53 (51,5)
ICD-10-kodiert?	Ja	45 (43,7)
	Nein	37 (35,9)
	Unvollständig	21 (20,4)
Konsil (psychiatrisch/psychosomatisch)	Ja	1 (1,0)
	Nein	102 (99,0)
Wiedervorstellung	Ja	59 (57,3)
	Nein	43 (41,7)
Verbleib	Stationär	41 (39,8)
	Ambulant	55 (53,4)
	Gegen ärztlichen Rat verlassen	7 (6,8)
Nationalität (häufigste)	Deutsch	70 (68,0)
	Polnisch	8 (7,8)
Wohnort (Postleitzahlen; häufigste)	Postleitzahlbezirk 10XXX	32 (31,1)
	Postleitzahlbezirk 13XXX	23 (22,3)
	Keine Angabe/ unbekannt	21 (20,4)
	Nicht Berlin	17 (16,5)

*ICD* International Classification of Disease

Bei einem der 103 psychosozialen Notfälle wurde im EH-Schein ein psychiatrisches Konsil eingefordert. Mehr als die Hälfte aller psychosozialen Notfälle wurde ambulant behandelt (53,4 %), 39,8 % wurden stationär aufgenommen. Sieben Patient*innen (6,8 %) verließen die zentrale Notaufnahme gegen ärztlichen Rat.

Der größte Teil der Patient*innen hatte eine deutsche Nationalität (68,0 %), gefolgt von wenigen Patient*innen mit polnischer (7,8 %) und unbekannter Nationalität (5,8 %). Die anderen Nationalitäten verteilten sich auf unterschiedliche Staaten.

Die erfassten Postleitzahlen entsprachen zu einem großen Teil dem Einzugsgebiet der zentralen Notaufnahme Charité Berlin Mitte (31,1 %; d. h. überwiegend dem Postleitzahlbezirk 10) und in 22,3 % der Fälle dem Postleitzahlbezirk 13. Überdies kamen einige Patient*innen (16,5 %) von außerhalb des Einzugsgebiets. Bei 20,4 % der Patient*innen waren keine Postleitzahlen angegeben, unter diesen befanden sich auch Personen ohne festen Wohnsitz (15,5 %).

## Diskussion

Die Untersuchung der Notfälle innerhalb einer Woche in der zentralen Notaufnahme der Charité Campus Mitte zeigte einen Anteil von 11,9 % an psychosozialen bzw. von 12,9 % an psychosozialen und/oder psychiatrischen Notfällen, die retrospektiv in der klinischen Routinedokumentation identifiziert werden konnten. Der ermittelte Anteil der psychosozialen Notfälle unter den Notaufnahmepatient*innen liegt im Bereich der in früheren Arbeiten ermittelten Häufigkeiten von 11,5 % [24] bis zu 14–20 % [11]. Allerdings beziehen sich diese Arbeiten auf Patient*innen mit einem rettungsdienstlichen Einsatz.

Die Dunkelziffer an psychosozialen Notfällen (55,9 %), die aus der Routinedokumentation identifiziert wurden, jedoch nicht (35,9 %) oder nicht vollständig (20,4 %) aus den kodierten Diagnosen abgeleitet werden konnten, war sehr hoch. Die hohe Dunkelziffer psychosozialer Behandlungsanlässe in diagnosebasierten Routinedatenauswertungen (Abrechnungsdaten, Krankenausstatistik) lässt darauf schließen, dass diese Kodierungen nur sehr limitierten Aufschluss über den tatsächlichen Anteil dieser Fälle in Notaufnahmedaten liefern können. Daher ist mit einem noch höheren Anteil psychosozialer Behandlungsanlässe bei einem prospektiven Screening zu rechnen.

In der untersuchten Population zeigte sich ein signifikant höherer Anteil an Männern unter den psychosozialen Notfällen im Vergleich zu nichtpsychosozialen Notfällen. Im Gegensatz zur vorliegenden Studie konnte in einer weiteren Studie jedoch kein signifikanter Unterschied in der Geschlechterverteilung zwischen Patient*innen mit psychosozialem Notfall und anderen Patient*innen gezeigt werden [24]. Bezüglich der Altersverteilung zeigte sich in unserer Studie kein signifikanter Unterschied zwischen den psychosozialen Notfällen und anderen Patient*innenfällen in der Notaufnahme. In einer Studie von Schmitt et al., in der Notarztprotokolle ausgewertet wurden, überwog unter den psychosozialen und psychiatrischen Notfällen der Anteil der Altersgruppe der 30- bis 50-Jährigen, unter den restlichen Notarzteinsätzen hingegen der über 60-Jährigen [24]. Auch in der vorliegenden Arbeit zeigt sich, dass die Gruppe der psychosozialen Notfälle eher jünger ist, jedoch konnte hier kein signifikanter Unterschied festgestellt werden.

Die häufigsten Behandlungsanlässe unter den psychosozialen Notfällen stellten Alkohol- und Substanzmissbrauch, gefolgt von Obdachlosigkeit, akuter Belastungsreaktion bzw. Überforderung, Angst- und Panikstörungen und Verwahrlosung dar. In über der Hälfte der psychosozialen Notfälle kam es zur Wiedervorstellung in der Notaufnahme mit erneut psychosozialem Behandlungsanlass. Aus weiteren statistischen Erhebungen ergab sich, dass von Obdachlosigkeit eher Männer betroffen sind [11]. Das konnte für andere soziale Umstände wie Arbeitslosigkeit nicht gezeigt werden [21]. Daher könnten sich diese Unterschiede in der Geschlechterverteilung aus der verwendeten Definition für einen psychosozialen Notfall ergeben, da beispielsweise Obdachlosigkeit in der vorliegenden Arbeit als psychosozialer Notfall gewertet wurde, Arbeitslosigkeit als alleiniger Umstand jedoch nicht. In einigen Studien zu rein psychiatrischen Notfällen gab es wiederum erhöhte Männeranteile [8, 13]. In einer weiteren Studie von Sefrin und Ripberger, in der psychische und soziale Umstände getrennt ausgewertet wurden, war unter den psychischen Notfällen Substanzmissbrauch ebenfalls der häufigste Behandlungsanlass [25].

In früheren Studien konnte gezeigt werden, dass verminderter sozialer Rückhalt und psychosoziale Belastungsfaktoren für häufige Wiedervorstellungen in Notaufnahmen prädisponieren [2]. Häufig werden psychosoziale Behandlungsanlässe auch im Zusammenhang mit sog. Frequent Users, d. h. häufigen Notaufnahmenutzer*innen [6]. festgestellt [4, 5, 20, 26]. Die vorliegende Studie bestätigt einen hohen Anteil erfasster Wiedervorstellungen (57,3 %) unter den psychosozialen Notfällen.

Im Vergleich zu der Verteilung von ausländischen Staatsangehörigkeiten unter den Einwohner*innen Berlins sind in dieser Studie Einwohner*innen mit deutscher Staatsangehörigkeit überrepräsentiert (Berlin-Brandenburg 2016; [3]). Dies widerspricht in Teilen den Erkenntnissen aus anderen Studien, nach denen Menschen mit Migrationshintergrund beispielsweise eine höhere Inanspruchnahme von Notaufnahmen unter anderem auch für psychiatrische Behandlungsanlässe aufzeigen [12, 19]. Allerdings wurde aus den Daten nur die Staatsangehörigkeit erhoben, die zu einer Untererfassung des Migrationshintergrunds führen kann.

### Limitationen der Arbeit

Unter dem Begriff „psychosoziale Notfälle“ wurden im Rahmen dieser Arbeit bestimmte Behandlungsanlässe definiert, die aus der Literatur abgeleitet wurden. Die Begriffsdefinition und die Differenzierung zwischen psychischen, psychiatrischen und sozialen Behandlungsanlässen ist in der vorliegenden Literatur heterogen und konnte im Rahmen dieser retrospektiven Studie nicht adäquat erhoben werden. Daher müssen die Ergebnisse dieser Studie, wie auch andere Arbeiten zum Thema psychosoziale Notfälle, in Relation zu der zugrunde liegenden Begriffsdefinition dieser Studie interpretiert werden. Für zukünftige, prospektive Projekte sollte eine Unterscheidung der verschiedenen Entitäten und eine Goldstandardbeurteilung der Behandlungsanlässe durch ein entsprechend interdisziplinäres Panel angestrebt werden.

Die Limitationen der Arbeit ergeben sich weiterhin aus dem retrospektiven, unizentrischen Studiendesign. Aufgrund des retrospektiven Ansatzes konnte kein direkter Einfluss auf die Datenerfassung und -dokumentation in der Notaufnahme genommen werden. Wie bereits diskutiert führt dies vermutlich zu einer Unterschätzung der Prävalenz psychosozialer Behandlungsanlässe in der Notaufnahme. Mit einer prospektiven Studie und standardisierten Screeningtools könnte dem entgegengewirkt werden.

Das unizentrische Design beinhaltet zusätzlich die Limitation, dass lediglich die Sozialstruktur eines Standorts und dessen Einzugsgebiet einbezogen werden konnte. Daher können die Daten nur bedingt als repräsentativ eingeschätzt werden, was unter anderem auch die Überrepräsentativität deutscher Staatsangehörigkeiten (am Standort Mitte) im Vergleich zur Gesamtbevölkerung Berlins erklären könnte. Gleichzeitig leistet die Studie trotz des unizentrischen Ansatzes einen entscheidenden Beitrag zur bislang mangelnden Quantifizierung psychosozialer Notfälle. Die sich aus den Daten ergebende Annahme einer hohen Dunkelziffer verdeutlicht, dass eine Erhebung psychosozialer Notfälle über diagnostische Routinedaten zwar multizentrisch leicht umsetzbar wäre, aber zu einer starken Unterschätzung der eigentlichen Prävalenz psychosozialer Notfälle führen würde.

### Schlussfolgerungen

Erstmalig konnte für eine Notaufnahme in Deutschland gezeigt werden, dass es einen relevanten Anteil psychosozialer Begleit- oder Hauptbehandlungsanlässe in der Notaufnahme gibt, die zu einem überwiegenden Teil in den kodierten Diagnosen nicht identifizierbar sind. Es ist davon auszugehen, dass die Dunkelziffer noch höher liegt. Notaufnahmen stellen somit eine wichtige Anlaufstelle für vulnerable Patient*innengruppen dar, die dort bisher weder ausreichend identifiziert noch bedarfsgerecht behandelt werden. Forschungsbedarf ergibt sich damit vor allem hinsichtlich einer klaren Begriffsdefinition des „psychosozialen Notfalls“, dessen prospektiver Identifikation und der Entwicklung von Interventionen, um Wiedervorstellungen zu vermeiden und die Patient*innen adäquat zu versorgen oder in bedarfsgerechte Strukturen weiterzuleiten.

## Fazit für die Praxis

- Bisher fehlt eine einheitliche Definition des psychosozialen Notfalls im notfallmedizinischen Kontext.
- Es findet sich ein relevanter Anteil psychosozialer Behandlungsanlässe in den Routinedaten zur Notfallversorgung in der Notaufnahme.
- Der Anteil der nicht in den Diagnosen kodierten Fälle ist hoch.
- Es ist eine hohe Dunkelziffer an nichtkodierten Fällen zu erwarten, die durch prospektive Forschungsansätze adressiert werden muss.
